# The Pivotal Player: Components of NF-κB Pathway as Promising Biomarkers in Colorectal Cancer

**DOI:** 10.3390/ijms22147429

**Published:** 2021-07-11

**Authors:** Matthew Martin, Mengyao Sun, Aishat Motolani, Tao Lu

**Affiliations:** 1 Department of Pharmacology and Toxicology, Indiana University School of Medicine, 635 Barnhill Drive, Indianapolis, IN 46202, USA; mm217@iu.edu (M.M.); sun19@iu.edu (M.S.); amotolan@iu.edu (A.M.); 2 Department of Biochemistry and Molecular Biology, Indiana University School of Medicine, 635 Barnhill Drive, Indianapolis, IN 46202, USA; 3 Department of Medical and Molecular Genetics, Indiana University School of Medicine, 975 West Walnut Street, Indianapolis, IN 46202, USA

**Keywords:** biomarker, colorectal cancer, NF-κB, therapeutics, transcription factor

## Abstract

Over the last several decades, colorectal cancer (CRC) has been one of the most prevalent cancers. While significant progress has been made in both diagnostic screening and therapeutic approaches, a large knowledge gap still remains regarding the early identification and treatment of CRC. Specifically, identification of CRC biomarkers that can help with the creation of targeted therapies as well as increasing the ability for clinicians to predict the biological response of a patient to therapeutics, is of particular importance. This review provides an overview of CRC and its progression stages, as well as the basic types of CRC biomarkers. We then lay out the synopsis of signaling pathways related to CRC, and further highlight the pivotal and multifaceted role of nuclear factor (NF) κB signaling in CRC. Particularly, we bring forth knowledge regarding the tumor microenvironment (TME) in CRC, and its complex interaction with cancer cells. We also provide examples of NF-κB signaling-related CRC biomarkers, and ongoing efforts made at targeting NF-κB signaling in CRC treatment. We conclude and anticipate that with more emerging novel regulators of the NF-κB pathway being discovered, together with their in-depth characterization and the integration of large groups of genomic, transcriptomic and proteomic data, the day of successful development of more ideal NF-κB inhibitors is fast approaching.

## 1. Introduction

### 1.1. Brief View of Colorectal Cancer

From the Centers for Disease Control and Prevention (CDC), colorectal cancer (CRC), also known as bowel cancer, refers to cancers that originate in the colon or rectum. This is further defined by the positioning of the cancer, dictating whether it is termed colon cancer or rectum cancer.

Decades ago, colorectal cancer was rarely diagnosed. However, now it is one of the leading carcinomas found in western countries and the fourth most lethal cancer worldwide [1,2,3]. CRC is the third most common malignancy in men, ranked prior to prostate cancer, and the second predominant malignancy in women, right after breast cancer [3]. Its morbidity increases with age, especially for people over 50 years old. The average age of onset is around 70 years old. In addition to aging, the development of CRC is also influenced by many other factors related to a low fiber diet, lack of physical exercise, obesity, smoking and other environmental factors. This type of CRC represents ~70% of total cases and constitutes the sporadic type of CRC [4,5]. Besides that, a minority of cases report the development of CRC at a much younger age of 25–30 years old, and their incidence is attributed to hereditary factors, accounting for the remaining 30% of cases. These make up the hereditary non-polyposis type of CRC, also called Lynch syndrome [3,4].

**Figure 1:**
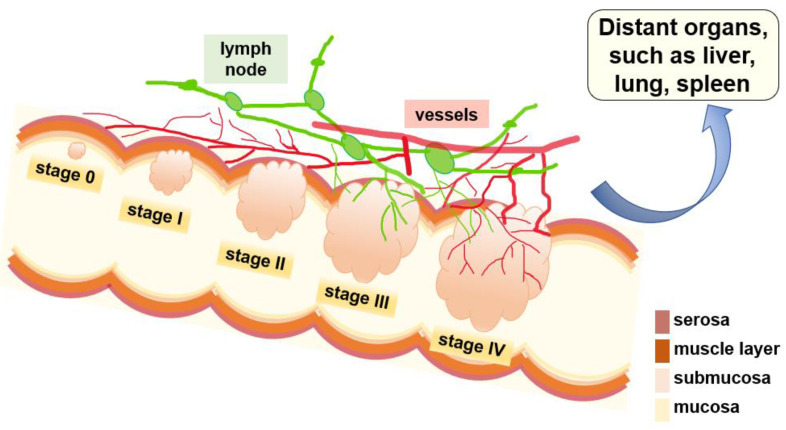
Stages of CRC development. *Stage 0* is the earliest stage, also known as carcinoma in situ, with polyps only present in the inner layer of the colon or rectum. In *Stage I*, the tumor has passed the inner layer of the colon or rectum but has not exceeded the wall of the rectum or colon. In *Stage II*, the cancer has grown into the wall of the colon or rectum but not yet reached nearby lymph nodes. In *Stage III*, the cancer has invaded nearby lymph nodes but is not present in the other organs. In *Stage IV*, the cancer aggresses distant organs of the body, such as the liver or lungs. (Adapted from [17].)

As illustrated in Figure 1, CRC develops in a step-wise manner, starting from precancerous polyps, the earliest stage of CRC and numbered “0”, to the other four main stages, which are numbered I–IV and defined based on tumor size, depth the tumor has grown into the tissue, degree of invasion and metastasis. For instance, as shown in Figure 1, at *stage 0*, aberrant cells grow up in the innermost layer (mucosa) and form tubercules in the colon or rectum, but these cells are non-cancerous [6,7,8]. At *stage I*, the tumor has passed the inner layer of the colon or rectum but has not exceeded the wall of the rectum or colon. At *stage II*, the cancer has grown into the wall of the colon or rectum but not yet reached nearby lymph nodes [9,10,11,12,13,14,15,16]. If the tumors further progress and invade the nearby lymph nodes, they enter *stage III*. Then, if the tumor cells aggressively diffuse into the lymphatic and vascular pathways and eventually to the distant organs, they have progressed to the advanced *stage IV*, which has a very poor prognosis and very high mortality.

### 1.2. Overview of Types of Biomarkers of CRC

Biomarkers play an important role in the early diagnosis and targeted treatment of CRC [17]. Biomarkers of CRC can be widely characterized into two major groups: *diagnostic* and *clinical biomarkers*. *Diagnostic biomarkers* can act as an early detection or suggestion of disease, while *clinical biomarkers* can be further classified as *predictive* or *prognostic biomarkers*.

#### 1.2.1. CRC Diagnostic Biomarker Examples

An example of a *diagnostic biomarker* is the case of multiple tumor suppressor 1 (MTS1), which was identified as a protein downregulated in CRC patients and as a marker for metastasis [18]. While initial biomarkers were discovered in blood tests of patients, several other avenues of testing have shown promise for identification and diagnosis of CRC. These include further blood-based biomarkers, such as CA 19–9, tissue polypeptide-specific antigen (TPS) and tissue polypeptide antigen (TPA), as well as cytokeratins 8, 18 and 19 [19,20]. Other types of biomarker assay methods include testing levels of cell-free DNA (cfDNA) to determine cellular apoptosis in CRC patients [21], and stool-based tests. Due to the connection of CRC to gastrointestinal systems, the Guaiac-based fecal occult blood test (gFOBT) has been used as a screening tool for CRC since the 1990s, which can detect the peroxidase reaction of hemoglobin in the feces. However, this technique is highly diet-reliant and low in sensitivity. Another stool-based test is the fecal immunochemical test (FIT), which detects hemoglobin with specific antibodies, but has shown issues with detection based on the original site of the cancer, as well as the time at which samples were tested [22]. Besides testing of physical stool samples for hemoglobin, there have been great strides in DNA and RNA-based stool tests [23]. These tests are less invasive for patients and somewhat less time-dependent. MiRNA in stools has also been tested and determined as a feasible biomarker for CRC [24]. Furthermore, protein-based stool and blood markers are also feasible tools for the identification and treatment of CRC. These include fecal calprotectin and M2 pyruvate kinase, both of which have their own difficulties for the identification of CRC [25]. Calprotectin has been shown to have non-specific expression in non-cancer-related inflammation, while M2 pyruvate kinase has shown decreased sensitivity in CRC, and thus is not always considered a strong biomarker for CRC. These types of biomarkers are only a few examples of those that have historically been used to identify the presence of CRC in patients.

#### 1.2.2. CRC Clinical Biomarker Examples

Among *clinical biomarkers*, the subcategory of *predictive biomarkers* refers to biomarkers that can indicate response to specific treatments or therapies, while *prognostic biomarkers* indicate the response of a patient independent of therapy [26]. An example of a *predictive biomarker* is poly(C)-binding protein 1 (PCBP1), which was shown to be highly elevated in patients with a resistance to the therapy of oxaliplatin, indicating a role of this protein in therapeutic resistance in CRC [27]. *Prognostic biomarkers* include markers independent of CRC, such as carcinoembryonic antigen (CEA), which was the first biomarker discovered in CRC in 1965 [28]. CEA has been used as a *prognostic biomarker* for the presence of CRC for many years despite concerns of elevated CEA levels in patients with inflammatory bowel disease and other inflammatory diseases [29].

The difficulty remains that while some of these biomarkers are useful for prediction and some biomarkers are useful for guiding therapeutic applications for treatment of CRC, there still remains a large knowledge gap between biomarkers identified by basic scientific approaches and connecting those novel discoveries to earlier discovery and treatment of CRC.

### 1.3. Synopsis of Signaling Pathways Related to CRC

CRC progression can be regulated through numerous signaling pathways. Dysregulation of these signaling pathways plays a critical role in CRC through promotion of cell proliferation and migration, as well as inhibiting apoptosis and cell death pathways through a multitude of interactions and signal feedback loops [30]. Some of these include the Wingless and Int-1 (WNT) signaling pathway, phosphatidylinositol 3-kinase/protein kinase B (PI3K/Akt) pathway, Hedgehog pathway, epidermal growth factor receptor (ErbB) pathway, Ras homolog family member A (RHOA) pathway, mitogen-activated protein kinase (MAPK) pathway and c-Jun N-terminal kinase (JNK) pathway [31,32,33,34,35,36,37,38]. CRC regulation by different signaling pathways can be complicated and interconnected [30]. There is some evidence of crosstalk between these signaling pathways and also with a critical signaling pathway’s nuclear factor κB (NF-κB). For example, Wnt signaling has been shown to impact Notch, JNK and Ras signaling [39]. ErbB can regulate the PI3K/Akt, Wnt and Ras signaling pathways [36]. Additionally, a mutant Kirsten rat sarcoma viral oncogene homolog (KRAS) protein can increase therapeutic resistance to ErbB-targeted drugs, such as cetuximab or panizumab [40]. PI3K/AKT activation can also promote NF-κB activation [41]. The overall crosstalk between these signaling pathways is tightly regulated by several factors, including glycogen synthase kinase 3 beta (GSK3β), p53, phosphatase and tensin homolog (PTEN) and KRAS. All of these are of critical importance in CRC development. These are just a few of the possible crosstalk interactions between signaling pathways involved in CRC development and progression. These interactions and overall dysregulation of signaling pathways in CRC can lead to increased tumorigenesis as well as increasing the so-called “hallmarks of cancer” [42].

Since many of the aforementioned signals are well-reviewed in ample other publications [42], in this review, we will mainly focus on the role of one of the most important signals, i.e., NF-κB signaling, as detailed below.

## 2. NF-κB Signaling and Its Important Role in CRC

### 2.1. NF-κB Signaling

**Figure 2:**
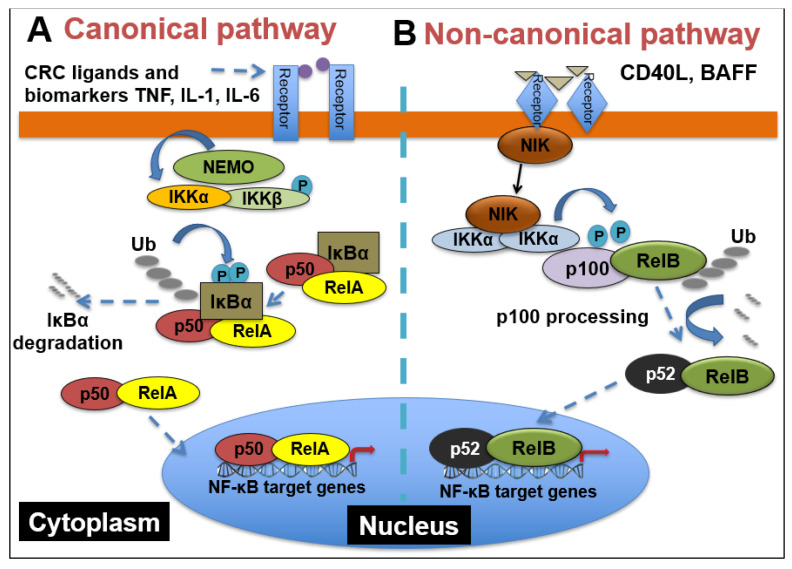
NF-κB signaling pathways. (**A**) Canonical NF-κB receptors TNFR, IL-1R and TLR are activated by their respective ligands, resulting in IKKβ activation. CRC biomarkers such as IL-6, IL-1 and TNFα can enhance this activity. Activated IKKβ phosphorylates IκBα, which causes its subsequent ubiquitination, separation from the p65/p50 complex and proteasomal degradation. p65/p50 heterodimers then translocate to the nucleus and can bind to their respective DNA elements and promote gene transcription. (**B**) In the noncanonical NF-kB signaling pathway, activation of noncanonical NF-κB receptors, including RANK and CD40, activates NIK, resulting in IKKα phosphorylation. Activated IKKα then phosphorylates p100, causing its polyubiquitination and processing to p52 via proteasomal degradation. The p52/RelB heterodimer then translocates to the nucleus to bind to its respective DNA elements.

NF-κB comprises a family of five transcription factors that regulate the expression of a variety of genes involved in several biological processes. These processes include inflammation, cellular development and differentiation, cell cycle progression, cell migration and so on [43]. The members of the NF-κB family include RelA/p65, RelB, c-Rel, NF-κB1(p50/p105) and NF- κB2(p52/p100), and they function as dimers in two separate but interconnected arms of the NF-κB pathway: the canonical pathway and the non-canonical pathway. As shown in Figure 2, in the canonical pathway, which is often activated by growth factors, lipopolysaccharides (LPS) and cytokines, the inhibitor of κB kinaseβ (IKKβ) in the IKK complex is phosphorylated. Phosphorylated IKKβ triggers a signaling cascade that results in the phosphorylation of IκBα and its subsequent degradation, causing the translocation of p65 and p50 heterodimers into the nucleus [44]. The binding of p65/p50 dimers to their cognate κB motif leads to the expression of NF-κB target genes. On the other hand, the non-canonical pathway, which is mainly involved in cell development, is activated by tumor necrosis factor receptor (TNFR) superfamily members’ ligands, including cluster of differentiation 40 ligand (CD40L), B-cell activating factor (BAFF), receptor activator of nuclear factor-κB ligand (RANKL) and lymphotoxin-β (LT-β) [45]. The stimulation of TNFR induces the phosphorylation of NF-κB-inducing kinase (NIK), which further phosphorylates IKKα. Then, activated IKKα induces the phosphorylation of p100, triggering its proteasomal processing to p52. Following the transformation of p100 to p52, the p52/RelB dimer undergoes nuclear translocation to promote gene transcription [46].

### 2.2. Myriad Functions of NF-κB Signaling and Complex Interactions between Cancer Cells and the Tumor Microenvironment (TME) in CRC

**Figure 3:**
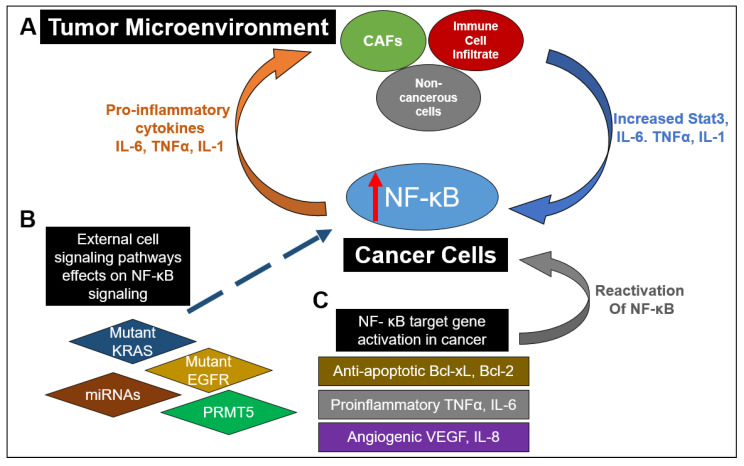
The myriad and varied interactions between NF-κB signaling in cancer and the tumor microenvironment (TME). (**A**) Increased NF-κB activation in cancer cells can result in the release of proinflammatory cytokines into the TME. This results in stimulation of cancer-associated fibroblasts (CAFs), immune cell infiltrates (such as macrophages) and noncancerous cells to release IL-6 and TNFα, as well as promoting STAT3 activation. Release of these factors back into the TME further enhances NF-κB activation in cancer cells. (**B**) Mutations of proteins in other pathways or lack of expression of those proteins have been shown to enhance NF-κB activation. These include mutant EGFR and KRAS in the RAS-RAF signaling pathway as well as miRNAs in numerous signaling pathways. Dysregulation of key regulators in NF-κB signaling such as PRMT5 can also contribute to upregulation of NF-κB activity. (**C**) NF-κB downstream target genes can act to promote a proinflammatory microenvironment by promoting the release of antiapoptotic factors such as Bcl-xL and Bcl-2, proinflammatory factors TNFα and IL-6, angiogenic factors VEGF and IL8 and proinflammatory chemokines. Furthermore, release of pro-inflammatory cytokines can restimulate NF-κB activation. All these factors contribute to a continual stimulation of NF-κB signaling and an increasingly pro-inflammatory environment.

As a major nexus of inflammation and cancer, NF-κB signaling has been reported to be extensively implicated in CRC progression. From the formation of polyps to the development of an invasive adenocarcinoma, NF-κB has been shown to play a role in multiple stages of malignancy development in the colon [47]. Interestingly, the aberrant activation of NF-κB has been reported in about 50% of CRC patients, and it has also been shown to promote the development of colitis-associated cancer [48]. As depicted in Figure 3, this constitutive activation of NF-κB drives the establishment of a pro-inflammatory tumor microenvironment in CRC, which feeds into multiple cancer hallmarks, including increased cell survival, proliferation, metastasis and angiogenesis. For example, in CRC, NF-κB upregulates the expression of anti-apoptotic proteins like Bcl-2-associated athanogene-1(BAG-1), B-cell lymphoma 2 (Bcl-2) and B-cell lymphoma-extra-large (Bcl-xL) proteins. Similarly, enhanced NF-κB function in CRC promotes he expression of proinflammatory cytokines such as TNFα, IL-6 and IL-1β, increases the levels of angiogenic factors like HIF-1α, IL-8 and VEGF and facilitates the expression of metastatic genes, including several chemokines, cytoskeletal genes and matrix metalloproteinases (MMPs) [49]. Moreover, as governed by dysregulated upstream proteins, such as protein arginine methyltransferase 5 (PRMT5), NF-κB has been shown to contribute to CRC cell growth, anchorage-independent growth and cell migration [50,51,52,53]. NF-κB also hampers the effectiveness of current CRC chemotherapeutic drugs via upregulation of anti-apoptotic proteins and chemokines [47].

Besides these factors, NF-κB also plays a critical role in the tumor microenvironment (TME) of cancer cells (Figure 3). Specifically in the context of CRC, IL-6 and TNFα act to mediate inflammation and cancer in the TME [54,55,56]. IL-6 has been shown to be increased in the tumor tissue and serum of patients with CRC and is correlated with lower patient survival [57]. TNFα has also been shown to have high serum expression in CRC patients and is linked with poor prognosis [58]. This high expression of IL-6 and TNFα promotes invasion, metastasis, angiogenesis, and therapeutic resistance [59,60]. NF-κB activation can result in activation of Stat3, which can lead to greater interactions and communication between cancer cells and the TME [61]. Interactions between cancer cells and the TME are critical for maintenance of tumor growth [61]. Additionally, immune cell infiltrates of tumor-associated macrophages can produce IL-6 and TNFα in CRC [62]. Another important component of the TME are cancer stromal fibroblasts (CAFs), which in CRC, also produce IL-6, further enhancing the interaction with the TME [60]. These data suggest the critical importance and complex nature of NF-κB signaling in relation to signaling within the TME between cancer cells and noncancerous cells in the microenvironment.

Collectively, these studies demonstrate the all-encompassing role that NF-κB plays in CRC progression and the potential it holds as a viable target for CRC treatment.

## 3. NF-κB Signaling-Related CRC Biomarkers

The prototypical NF-κB is the p65/p50 heterodimer. Both subunits have been suggested as CRC biomarkers. As shown in Table 1, p65 overexpression in CRC tissue has been correlated with increasing tumor staging and decreased overall survival [63]. In one study, NF-κB expression in patients who had stage-III CRC tumors resected was identified as an independent indicator of patient survival. In addition to the p65, the p50 subunit of NF-κB also plays an important role in CRC (Table 1). For instance, in a radiotherapy study for rectal cancer, NF-κB activation resulted in NF-κB target genes’ upregulation and increased cell survival [64], where the p50 subunit served as a prognostic biomarker for overall survival [64]. In addition to radiotherapy, hyperactivation of NF-κB has been linked to chemoresistance in CRC [65,66]. For example, NF-κB hyperactivation led to therapeutic resistance to chemo drugs, such as fluorouracil or 5-FU, oxaliplatin and irinotecan. In particular, irinotecan resistance through NF-κB hyperactivation is a difficulty for later-stage metastatic CRC [67,68]. These results indicate the importance of NF-κB subunits p65 and p50, as both diagnostic and prognostic biomarkers of CRC.

Apart from NF-κB itself, other biomarkers linked to NF-κB signaling include the mutational status of KRAS (Table 1). That is determined in part because it affects treatment with anti-epidermal growth factor receptor (EGFR) monoclonal antibodies which are commonly used for metastatic CRC treatment [69]. Since there is crosstalk between NF-κB and the RAS-RAF signaling pathway, oncogenic KRAS can cause NF-κB activation [69] (Figure 3). Unsurprisingly, knockdown of KRAS reduces p65 expression in CRC cells, and patients with KRAS mutations also have been shown to have higher p65 expression [70]. Overall, this evidence indicates that NF-κB activation due to KRAS mutations decreases survival and chemotherapeutic response for patients with metastatic CRC [70]. Importantly, one study in CRC patients with wild-type KRAS treated with irinotecan and monoclonal antibodies for EGFR showed tumors highly expressing p65 had less survival and chemotherapeutic response than patients with NF-κB negative tumors, suggesting the critical importance of NF-κB expression in CRC prognosis, independent of KRAS [70].

Recently, some new biomarkers were identified in basic scientific settings. As summarized in Table 1, for instance, microRNA 21 (miR-21) was identified as a positive regulator of Ki-67 and promoter of NF-κB activity in colitis-associated CRC mouse models [71]. Other targets were the combination of NF-κB-interacting lncRNA (NKILA) microRNA 103 (miR-103) and microRNA 107 (miR-107), which were shown to be upregulated or downregulated in CRC compared to normal tissue [72]. Another biomarker was protein forkhead box K2 (FOXK2), which was found to be upregulated in metastatic CRC tissues [73]. Interestingly, the P2 × 7 receptor (P2 × 7R) has been shown to be upregulated in CRC tissue and triggers cellular proliferation and invasion through hyper NF-κB activity [74]. Additionally, high growth arrest and DNA damage-inducible beta (GADD45B) expression in CRC has been shown to be linked to poor prognosis and therapeutic response through increased NF-κB activity [75]. It will not be surprising if more candidate biomarkers continue to emerge, some of which will surely be explored for their potential application as CRC biomarkers in clinical settings in the future.

## 4. Effort on Targeting NF-κB Signaling in CRC Treatment and Conclusive Remarks

The aforementioned evidence strongly supports the notion of targeting the NF-κB pathway as an important approach for CRC therapy. However, since NF-κB is a master player in the immune response, direct inhibition of NF-κB may lead to unwanted immunosuppression. Though this side effect could be partially overcome by more frequent and low-dosage treatment, targeting regulators instead of NF-κB itself is still a better option for CRC treatment.

In fact, some effort is already underway for the development of inhibitors of the NF-κB signaling pathway in CRC treatment. For instance, certain phytochemicals, such as curcumin, ginseng extract, resveratrol and green tea extract have shown beneficial effects in CRC patients by inhibiting IKK/NF-κB activity ([76,77]). C086 is a more potent compound derived from curcumin that can decrease NF-κB activity in both CRC cells and xenograft tumors [76]. Additionally, proteasome inhibitors, such as bortezomib, have demonstrated some success by blocking the degradation of IκB, thereby inhibiting the release of NF-κB heterodimers to the nucleus. [77]. Other NF-κB inhibiting agents such as nonsteroidal anti-inflammatory drugs (NSAIDs) are also used to lower the overall risk of colitis-associated CRC development [78,79]. Moreover, glucocorticoids such as dexamethasone are proven efficacious NF-κB inhibitors [80]; the combined use of a glucocorticoid with other therapeutics has been investigated via clinical trials.

In sum, this evidence affirms the inhibition of the NF-κB pathway as a promising therapeutic approach for CRC. Considering the important role of NF-κB, which is involved in many physiological processes, approaches that can avoid systemic NF-κB inhibition are more desirable. The development of nanotechnology for drug delivery has helped the use of minimal dosages of drugs, targeted to limited areas of the body [81]. Though these endeavors are encouraging, there is still high demand for the development of the more ideal NF-κB inhibitors, so they may target CRC cancer cells as well as the surrounding TME, with maximum efficacy and as few systemic side effects as possible for CRC patients. One such direction could focus on targeted therapy. For example, the aforementioned PRMT5 has become a hot therapeutic target in cancer. Our group [51,52] with *EQon Pharmaceuticals*, and other drugmakers such as *GlaxoSmithKline* (GSK) and *Johnson & Johnson*, have been developing PRMT5 inhibitors to treat cancers. So far, a small number of candidate compounds have entered the early stage of clinical trials.

We are confident that, with more emerging novel regulators of the NF-κB pathway being discovered, together with the in-depth characterization of these newly identified biomarkers of CRC diagnosis and therapeutic response, along with the integration of large groups of genomic, transcriptomic and proteomic data, the goal of successful development of the most ideal NF-κB inhibitors will ultimately be achieved in the near future.

## Figures and Tables

**Table 1:** NF-κB-related CRC biomarkers.

Symbol	Description	Type of Biomarker (Diagnostic or Clinical)	References
p65	P65 subunit of NF-κB	Diagnostic	[63,65,66,67,68,69]
p50	P50 subunit of NF-κB	Clinical (radiation)	[64,67,68]
KRas	Kirsten rat sarcoma viral oncogene homolog	Clinical/diagnostic (chemotherapeutic)	[70]
mir-21	microRNA 21	Diagnostic	[71]
NKILA	NF-κB interacting lncRNA	Diagnostic	[72]
mir-103	microRNA 103	Diagnostic	[72]
mir-107	microRNA 107	Diagnostic	[72]
FOXK2	Protein forkhead box K2	Diagnostic	[73]
P2 × 7R	P2 × 7 receptor	Diagnostic	[74]
GADD45B	Growth arrest and DNA damage inducible Beta	Clinical/diagnostic (chemotherapeutic)	[75]

## Data Availability

Not applicable.
